# Genomic characterization of ependymomas reveals 6q loss as the most common aberration

**DOI:** 10.3892/or.2014.3271

**Published:** 2014-06-16

**Authors:** THALE KRISTIN OLSEN, LUDMILA GORUNOVA, TORSTEIN R. MELING, FRANCESCA MICCI, DAVID SCHEIE, BERNT DUE-TØNNESSEN, SVERRE HEIM, PETTER BRANDAL

**Affiliations:** 1 Section for Cancer Cytogenetics, Institute for Cancer Genetics and Informatics, Oslo University Hospital - The Norwegian Radium Hospital, Nydalen, 0424 Oslo, Norway; 2 Centre for Cancer Biomedicine, Faculty of Medicine, University of Oslo, The Norwegian Radium Hospital, Nydalen, 0424 Oslo, Norway; 3 Institute of Clinical Medicine, Faculty of Medicine, University of Oslo, Blindern, 0316 Oslo, Norway; 4 Department of Neurosurgery, Oslo University Hospital - Rikshospitalet, Nydalen, 0424 Oslo, Norway; 5 Department of Pathology, Oslo University Hospital - Rikshospitalet, Nydalen, 0424 Oslo, Norway; 6 Department of Oncology, Oslo University Hospital - The Norwegian Radium Hospital, Nydalen, 0424 Oslo, Norway

**Keywords:** ependymoma, cytogenetics, karyotyping, comparative genomic hybridization

## Abstract

Ependymomas are rare tumors of the central nervous system (CNS). They are classified based on tumor histology and grade, but the prognostic value of the WHO grading system remains controversial. Treatment is mainly surgical and by radiation. An improved knowledge of ependymoma biology is important to elucidate the pathogenesis, to improve classification schemes, and to identify novel potential treatment targets. Only 113 ependymoma karyotypes with chromosome aberrations are registered in the Mitelman database. We present the first study of ependymoma genomes combining karyotyping and high resolution comparative genomic hybridization (HR-CGH). Nineteen tumor samples were collected from three pediatric and 15 adult patients treated at Oslo University Hospital between 2005 and 2012. Histological diagnoses included subependymoma and myxopapillary ependymoma (WHO grade I), ependymoma (WHO grade II) and anaplastic ependymoma (WHO grade III). Four tumors were intraspinal and 15 were intracranial. Seventeen samples were successfully karyotyped, HR-CGH analysis was undertaken on 17 samples, and 15 of 19 tumors were analyzed using both methods. Twelve tumors had karyotypic abnormalities, mostly gains or losses of whole chromosomes. Structural rearrangements were found in four tumors, in two of which 2p23 was identified as a breakpoint region. Twelve tumors displayed genomic imbalances by HR-CGH analysis with loss of material at 6q as the most common. 6q loss, which was detected by one or both methods in seven of 12 (58%) abnormal tumors, and 5p gain (observed in five tumors; 42%) were the most common genomic aberrations in this series.

## Introduction

Ependymomas are primary neuroepithelial tumors of the central nervous system (CNS) mimicking ependymal cell differentiation. Intraspinal ependymomas are more common in the adult patient population whereas children more often have intracranial tumors (1). Ependymomas are subgrouped into subependymomas (WHO grade I), myxopapillary ependymomas (WHO grade I), ependymomas (WHO grade II), and anaplastic ependymomas (WHO grade III) (2). They are rare with an incidence of ~2 per million inhabitants per year (3). Ependymomas of grades II and III constitute 2–5% (4–7) of all primary CNS neoplasms and only 1–3% of brain tumors in adults (6,8). However, they are the fourth most common CNS neoplasm in children, constituting 6–12% of all intracranial pediatric tumors. Within the pediatric population, more than a third of the patients are 4 years of age or younger (2,4,6).

Surgery is the mainstay of ependymoma treatment and gross total tumor resection (GTR) is an independent prognostic factor affecting both overall (OS) and progression-free (PFS) survival (2,6,8–10). GTR is only achieved in ~50–75% of patients, however (8–10). The 5-year relative survival of ependymoma patients is reported to be ~70% (11,12) and children tend to fare worse than adults (13,14). Patients with intraspinal tumors have a better prognosis than do those with intracranial tumors (2,6,13,15) and some studies indicate that supratentorial ependymomas carry a worse prognosis than infratentorial lesions (13,16–19). Grade I ependymomas have a relatively good prognosis (2) whereas the histologic distinction between ependymoma grade II and III is a controversial issue of unclear prognostic importance (2,3,15,20–22). Genetic and molecular characteristics of these tumors may prove to be more reliable prognostic markers (15,23).

Only 113 ependymomas (of 2,511 CNS tumors) are registered in the Mitelman Database of Chromosome Aberrations in Cancer (24). Approximately two-thirds of ependymomas are karyotypically abnormal (25) and most of these are near-diploid (24,26). Loss of chromosome 22 has been the most common aberration (25,27) reported in approximately a third of tumors with abnormal karyotypes, with structural abnormalities of chromosome 22 being reported in another 11% (3,24). In a large meta-analysis (23) comparing CGH results in adult and pediatric ependymomas, chromosome 22 was the most common site of genomic loss in both groups. 1q gain was significantly more frequent in the pediatric tumors and was the most common aberration overall in this group. Gains of chromosomes 7, 9 and 12 were significantly more common in adult tumors which also had a higher number of genomic imbalances than did pediatric ependymomas. Thus, the authors suggested that ependymomas of the two age groups are genetically distinct.

No previous studies have investigated the ependymoma genome using a combination of CGH and G-banding techniques. We describe genomic and chromosomal aberrations in a series of histologically heterogeneous ependymomas.

## Materials and methods

### Patients and tumor samples

The tumor samples used in the present study (19 specimens from 18 patients) were prospectively collected between January 2005 and December 2012. Clinical and pathological details are provided in Table I. All patients underwent surgery at the Department of Neurosurgery, Oslo University Hospital-Rikshospitalet and none of them had received chemotherapy or radiotherapy prior to surgery. Patient age ranged from eight months to 75 years at the time of the primary surgery. There were six female and 12 male patients, and all but three samples were taken from primary tumors. Both spinal and intracranial tumors were included, and all histopathological diagnoses were reviewed according to the WHO 2007 classification system (2). Progression was defined as tumor growth on magnetic resonance imaging (MRI) and recurrence was defined as the appearance of a new tumor by MRI. Progression-free survival (PFS) was defined as the time interval between initial surgery and radiologically proven tumor progression or recurrence. Overall survival (OS) was defined as the time interval between primary surgery and death.

### G-banding and karyotyping

All tumor samples were processed for cytogenetic analysis using standard methods as described by Mandahl (28). The chromosomes of the dividing cells were G-banded and a karyotype was established in accordance with ISCN 2009 (29). Karyotypes with four or fewer aberrations were defined as simple, whereas karyotypes with five or more aberrations were defined as complex.

### DNA extraction and high-resolution comparative genomic hybridization (HR-CGH)

DNA was extracted from the tumor samples using Maxwell 16 (Promega, Madison, WI, USA) (12 samples), standard phenol/chloroform method (four samples), or MagAttract DNA Mini M48 kit (Qiagen, Hilden, Germany). DNA sample quality and concentrations were measured and assessed using NanoVue Plus (GE Healthcare Life Sciences, Uppsala, Sweden).

CGH was performed as described by Kallioniemi *et al* (30) with modifications as published by Kirchhoff *et al* (31,32) and Kraggerud *et al* (33). Normal male human DNA (Promega) and the LoVo cell line (Sigma-Aldrich, St. Louis, MO, USA) were used as negative and positive controls. Inverted DAPI images were used to identify the chromosomes in 12 suitable metaphases of good quality, and the average green-to-red fluorochrome ratios with 99.5% confidence intervals were calculated along the length of each chromosome. In the cases where the G-banded karyotype was not near-diploid, a 95% confidence interval was used. These ratio profiles were compared with dynamic standard reference intervals as described by Ribeiro *et al* (34). Aberrations were recorded when the case and standard reference profiles, with their respective confidence intervals, did not overlap. A green-to-red ratio of 2.0 was defined as the threshold for amplifications. The short arms of acrocentric chromosomes (chromosome 13, 14, 15, 21 and 22) and the Y chromosome were not included in the analysis due to the known repetitive sequences in these chromosomal arms (30). The results of the HR-CGH analysis were described according to ISCN 2009 guidelines (29). We defined four or fewer chromosomes with imbalances as simple genomic changes, as opposed to complex genomic changes when five or more chromosomes were imbalanced.

### Study approval and ethics

The study was approved by the Norwegian Regional Research Ethics Committee (reference number S-06046). Written, informed consent was obtained from 15 participants. The remaining three patients were included post mortem following permission from the Norwegian Directorate of Health. All tumor samples were collected from an approved biobank.

## Results

**Figure 1:**
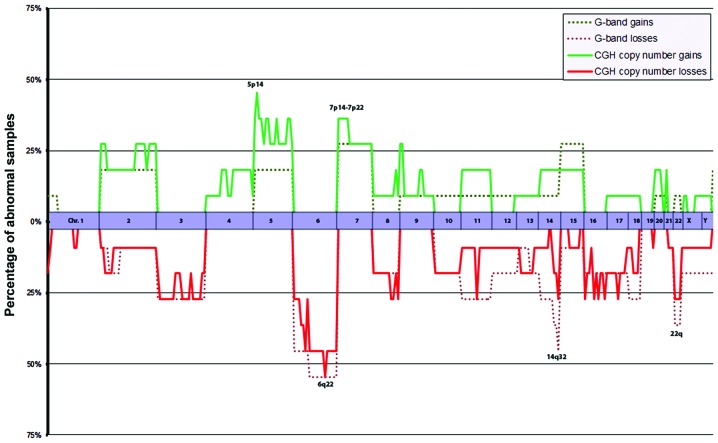
Gain and loss of genetic material by G-banding and HR-CGH in 18 ependymomas (sample 9-2 is not included in this graph). Green lines indicate gains, red lines indicate losses. The y-axis indicates the percentage of abnormal samples displaying certain aberrations; the x-axis indicates the chromosomes affected.

Karyotypes were established from 17 samples, the remaining two samples failed in culture. Tissue was available for DNA extraction and HR-CGH analysis for 17 of 19 samples. Fifteen samples (79%) were analyzed using both methods. The karyotypes and HR-CGH results are presented in Table II and Fig. 1. Tumor recurrence and/or progression occurred in six patients after an average of 3.1 years (median 1.6 years). Three patients died; one of postoperative complications, one of ependymoma, and one of other disease. GTR was achieved at primary surgery in 14 of 18 patients (78%). For samples 9-1 and 9-2, which were taken from the primary and recurrent tumor from one patient, GTR was achieved at both the initial surgery and at the time of recurrence. These two tumors are thoroughly described in a previous case report (35).

### G-banding and karyotyping

Twelve of 17 karyotypes (71%) were abnormal (Table II, Fig. 1): seven were near-diploid, three were near-triploid and two were near-tetraploid. Six (50%) karyotypes were simple, whereas the remaining six aberrant karyotypes had multiple chromosomal abnormalities. The number of aberrations in each abnormal sample ranged from one to 13. Eight of 12 (67%) abnormal samples displayed numerical aberrations only. Three samples (cases 3, 12 and 15) showed a combination of structural and numerical genomic abnormalities. One sample had an unbalanced t(2;14)(p23;q22) as its sole aberration (case 11). Three of the 12 abnormal karyotypes (25%) displayed a sole abnormality (cases 11, 17 and 18), but none of these were recurrent. None of the structural rearrangements occurred in more than one tumor. The most common numerical aberration was loss of chromosome 6, which was noted in six tumor samples, two of which were obtained from the same patient (primary tumor and recurrence; cases 9-1 and 9-2). Five tumors from four patients showed loss of chromosome 22. The overall gain/loss profile of 11 karyotypically abnormal tumor samples (Fig. 1; the recurrent tumor from the patient from whom we also had primary tumor material, case 9-1, is excluded) showed that the long arm of chromosome 6 was lost in six tumors (55%). This was mostly due to monosomy 6, but in one case because of an isochromosome 6p with loss of chromosome arm 6q. Material from 14q (14q24-qter) was lost in five (45%) samples; this was due to loss of the entire chromosome 14 in three tumors and to unbalanced structural rearrangements in two.

### HR-CGH analysis

HR-CGH analysis was informative in all 17 samples from which DNA was available. Twelve of 17 samples (71%) had genomic imbalances, and all analyzed chromosomes were affected in one or more samples. Gains were noted in nine and losses in 12 samples. No amplifications were found. The average number of aberrations per abnormal sample was 12, and gains (average 5.3) were slightly less common than losses (average 6.9). Five of 12 samples (42%) had simple genomic changes whereas the remaining seven (58%) were genomically complex with an average of 12 affected chromosomes per sample. The most common genomic imbalances in 11 tumor samples (Fig. 1; the recurrent tumor from the patient in which we also had material from the primary tumor, case 9-2, is again excluded) were copy number loss involving 6q (55%) and 6p (45%) and gain involving 5p (46%).

### Genomic aberrations in anatomical subgroups

Karyotypes were successfully obtained from three of four spinal tumors. One of them was normal (case 2) whereas the other two (cases 6 and 7) had near-triploid, complex karyotypes with numerical abnormalities only. HR-CGH was performed on three of four spinal lesions and all three were abnormal. Two tumors displayed complex genomic aberrations and all three had copy number losses at band 22q11.

Eight of nine (89%) infratentorial tumors were successfully karyotyped. Four of these were cytogenetically normal and one tumor (case 18) showed Y loss only. Two of the remaining three abnormal tumors (cases 4 and 17) had simple, near-diploid karyotypes with loss of chromosome 6. The last tumor (case 1) had a complex, near-triploid karyotype with several numerical aberrations. DNA material was available for HR-CGH analysis for eight of nine (89%) tumors. Three of these (38%; cases 1, 14 and 17) were abnormal, of which two harbored complex genomic imbalances. Genomic losses at 6p and 6q were found by HR-CGH in all three abnormal infratentorial tumors.

All six supratentorial tumors were karyotypically abnormal and in three of five (60%) tumors loss of material from 14q was noted. All four tumors harboring structural rearrangements were supratentorial. HR-CGH results from all six supratentorial samples were abnormal. When excluding the recurrent tumor (case 9-2), three of five (60%) cases displayed simple genomic changes.

### Genomic aberrations and tumor grade

Sixteen tumors were assessed; five grade I tumors (cases 2, 4, 8, 10 and 13), seven grade II tumors (cases 1, 5, 6, 7, 14, 17 and 18), and four grade III tumors (cases 3, 9-1, 12 and 16). Case 11 was not included due to its uncertain WHO grade and case 9-2 was not assessed because its corresponding primary tumor (case 9-1) was already included in the grade III group. Case 15 was excluded due to its histology (anaplastic ependymoma with prominent glioblastoma features).

Four of five (80%) grade I tumors were informative by G-banding. Three of these (75%) were karyotypically normal, whereas the remaining tumor (case 4) had a simple karyotype with monosomy 6. DNA was available for HR-CGH analysis in three of five tumors and all three were normal.

Six of seven grade II lesions were informative by G-banding. One of these (17%) had a normal karyotype. There were no structural chromosomal aberrations found in this group. The most common numerical aberrations were +7, +15 and −6 (three cases each; 50%). HR-CGH analyses were performed on all seven grade II ependymomas: one tumor (14%) did not harbor any genomic imbalances, two tumors (29%) displayed simple genomic imbalances, and four (57%) had complex HR-CGH profiles with an average of 17 aberrations per sample.

G-banded karyotypes were obtained from all four grade III ependymomas. One karyotype was normal, one tumor had a simple karyotype and two tumors had complex karyotypes. All four tumors were informative by HR-CGH. One of them had a normal profile, one had simple changes, whereas the remaining two tumors (cases 9-1 and 12) had complex profiles.

### Genomic aberrations and biological tumor behavior

Tumor samples from patients with known disease progression were compared to those with stable disease. Six patients were included (cases 3, 4, 9-1, 12, 14 and 16) in the progression group, eleven patients (cases 1, 2, 5, 6, 7, 8, 10, 11, 13, 15 and 18) in the ‘no progression’ group, whereas one patient (case 17) died of postoperative complications and was excluded. There were four grade III, one grade II, and one grade I tumor within the progression group.

The progression group was karyotypically heterogeneous. Two patients (33%) had a normal karyotype, two (cases 3 and 4) had karyotypes with simple changes, and the remaining two abnormal tumors were karyotypically complex. Aberrations leading to loss of 6q were found in three of four (75%) abnormal karyotypes. By HR-CGH, one tumor had a normal profile, one had a simple profile, and three tumors had complex changes. The most common imbalance by CGH was loss of material from band 6q22 which was noted in three of four (75%) abnormal samples.

The average follow-up for the 11 patients in the ‘no recurrence’ group was 5.4 years (median 6.1). Karyotypes were successfully obtained from nine of 11 (82%) tumors. Three of these were normal, three were karyotypically simple, while the remaining three samples had complex karyotypes. The most common karyotypic aberrations in this group were +7 and +15 as well as loss of material from the distal end of chromosome 14. These changes occurred in three tumors (50%) each. DNA was available for HR-CGH in all but one of the cases in this group of tumors. Four of them (40%) were normal. Among the six remaining cases there were three samples with simple and three samples with complex profiles. The most common imbalances were gains at 5p and 7p/7q as well as losses from 16p/16q and 22q, which were all found in three (50%) of the six abnormal samples.

## Discussion

**Figure 2:**
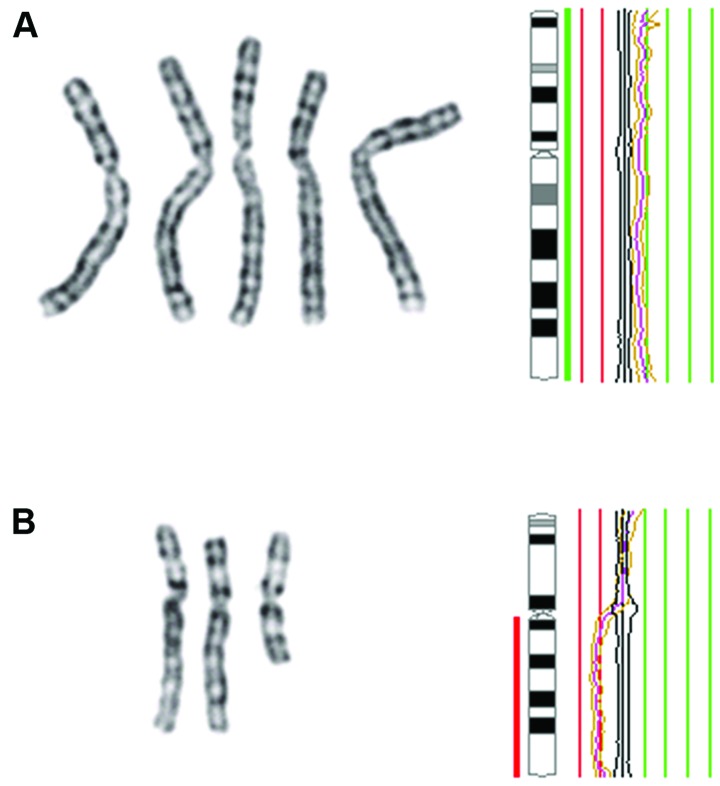
Case 3 had the karyotype 89–94, XXYY,+2, −6,i(6)(p10). G-banded chromosomes are shown to the left, corresponding CGH profiles to the right. In this case, G-banding provided additional information on tetraploidy and the mechanism of 6q loss. (A) Gain of chromosome 2. (B) Isochromosome 6p resulting in 6q loss.

There was good overall concordance between HR-CGH and G-banding results in the 19 ependymomas we examined (Figs. 1 and 2; Table II). Of the 15 tumor samples analyzed by both methods, three (20%) were normal by both G-banding and HR-CGH. Because cryptic balanced rearrangements are not visible in G-banded karyotypes and small genomic imbalances are below the HR-CGH resolution level, we cannot exclude the possibility that the observed normality was representative for the parenchyma in these tumors. At least equally likely is that there were not enough neoplastic cells present in these samples for the analytical techniques to pick up abnormalities. Some discrepancies between the two methods were observed, however; the most striking example of which was case 14. This tumor had a normal karyotype, but multiple gains and losses, mainly of whole chromosomes, when analyzed by HR-CGH. In this case, apparently the parenchymal cells did not divide *in vitro* and so remained undetected.

22q loss was previously reported to be the most common genomic aberration in ependymal tumors (23,25–27) and the present study confirmed this aberration to be a frequent finding (22%). However, the most common imbalance in the present series, irrespective of tumor histology, grade, or anatomical location, was 6q loss (39%). 6q loss was previously detected in ependymomas at a frequency of 15–30% (23,36–39). Some genes mapping to the long arm of chromosome 6 have previously been reported to be downregulated in ependymal tumors, including *SASH1* [a candidate tumor suppressor gene in breast (40) and colon (41) cancer], *TCP1* (involved in tubulin function), *ADM1* and *CDK11* (involved in cell proliferation) (15,42,43). According to the Cancer Gene Census (44), five genes located on chromosome 6 are involved in human cancers. Large-scale genomic alterations such as 6/6q loss might contribute to carcinogenesis by affecting these and/or presently unknown cancer genes; alternatively, the chromosomal aberration may lead to regulatory changes of an unknown type. Notably, Korshunov *et al* (38) proposed a risk stratification scheme for ependymomas based on cytogenetics with tumors harboring monosomy 6 (among other aberrations) being significantly associated with an excellent progression-free and overall survival. These findings were validated in an independent cohort of 170 patients. Some smaller studies have also investigated the impact of 6q deletions on prognosis. Monoranu *et al* (45) found that 6q25.3 deletions in pediatric, intracranial grade III ependymomas were significantly associated with longer overall survival. In contrast, Rajaram *et al* (46) found that 6q23 loss, detected by fluorescent *in situ* hybridization (FISH), was associated with disease progression in a mixed patient population. The present series is small and prognostic conclusions cannot be drawn. The potential of 6q loss as a prognostic marker is still in need of prospective evaluation in future studies (47). 5p gain is also a known abnormality in ependymomas and was detected in five of 12 tumors (42%) with copy number changes. Kilday *et al* (23) found that gain of chromosome 5 or chromosome arm 5p was reported in ~25% of adult ependymomas analyzed by CGH. In the present study, this aberration was not typical of any anatomical subgroup, but four of five (80%) tumors with 5p gain were grade II ependymomas.

Pediatric ependymomas appear to be genetically distinct from adult tumors; 1q gain was reported to be more common in children in spite of generally fewer genomic imbalances or even balanced genomic profiles by CGH (23,38,48). 1q gain was found to be associated with poor survival for intracranial ependymoma patients (38,47). In our series, 1q gain was not found in any tumor, neither adult nor pediatric. The three pediatric ependymomas all had simple karyotypes and few imbalances by HR-CGH. One of them was normal both by karyotyping and HR-CGH (case 16). Notably, this was a clinically aggressive tumor which progressed after 1.2 years in spite of active oncological treatment. Another pediatric tumor (case 15) was described by the neuropathologist as an anaplastic glioma with both astrocytic and ependymal features. The final diagnosis was ‘anaplastic glioma, WHO grade IV, most likely anaplastic ependymoma’ and the patient was treated according to ependymoma protocols. The karyotype was near-tetraploid but simple, with an interstitial 2p deletion, gain of chromosome 22, and loss of chromosome 10. The HR-CGH analysis confirmed the 2p deletion and also showed loss of 1p and 16q. Loss of chromosome 10 is a typical finding in glioblastomas (26,49) but was not frequent in the current ependymoma series. Furthermore, none of the most common aberrations in this series (Fig. 1), such as 6q loss, 5p gain, 7p/7q gain or 22q loss, were detected in this particular tumor. Glioblastomas most often have complex karyotypes with multiple structural and numeric abnormalities (49). Based on the cytogenetic findings, one might argue that this case bears more resemblance to astrocytomas/glioblastomas than to ependymomas.

Four tumors in this series (cases 3, 11, 12 and 15) harbored structural chromosomal rearrangements. Among these, case 11 is of particular interest. This tumor had a der(14)t(2;14) as its sole aberration by G-banding. The unbalanced translocation was reflected in the HR-CGH profile which displayed loss of material at 14q23qter but also gain of material at 2p23pter. Notably, this breakpoint region on chromosome 2 (2p23) was also involved in an interstitial 2p deletion found in another tumor (case 15). It is possible that the observed rearrangements affect putative cancer-related genes located in this chromosomal region. Thus, 2p23 is an area of interest for future molecular studies.

The present study is the first to combine karyotypic and CGH analyses of ependymomas. It provides an overview of the ependymoma genome and is a significant contribution to existing cytogenetic knowledge on these rare tumors. 6q loss and 5p gain were found to be the most common chromosomal aberrations in our heterogeneous patient population. The significance of these findings requires further analysis in larger studies.

## Figures and Tables

**Table I:** Clinical and pathological data of the 19 ependymoma samples from 18 patients.

Case no.	Gender/age (years)	Localization^a^	Histology^b^	WHO grade	Primary tumor/ recurrence	Extent of resection^c^	PFS/OS (years)	Current disease status^d^
1	F/48	IT	E (melanotic variant)	II	Primary	STR	8.5^e^	AWD
2	M/44	S	MPE	I	Primary	GTR	7.6^e^	NED
3	M/49	ST	AE	III	Recurrent	GTR	8.9	NED
4	M/69	IT	MPE	I	Recurrent	STR	4.6/5.8	DOD
5	F/47	S	E	II	Primary	GTR	7.5^e^	NED
6	M/42	S	E	II	Primary	GTR	6.9^e^	NED
7	M/53	S	E	II	Primary	GTR	6.5^e^	NED
8	M/61	IT	SE	I	Primary	GTR	6.1^e^	NED
9-1	M/38	ST	AE (giant cell type)	III	Primary	GTR	1.5	
9-2			AE	III	Recurrent	GTR	4.2^e^	NED
10	M/75	IT	SE	I	Primary	GTR	5.1^e^	NED
11	M/0.8	ST	E	II or III	Primary	GTR	5.1^e^	NED
12	M/42	ST	AE	III	Primary	GTR	0.8/1.3	DOE
13	F/46	IT	SE	I	Primary	GTR	3.4^e^	NED
14	F/46	IT	E	II	Primary	GTR	1.7	AWD
15	F/0.9	ST	AE/GBM	IV	Primary	STR	2.1^e^	AWD
16	F/1	IT	AE	III	Primary	STR	1.2	AWD
17	M/72	IT	E	II	Primary	GTR	0.0/0.0	DPC
18	M/67	IT	E with SE component	II	Primary	GTR	0.7^e^	NED

Samples 9-1 and 9-2 were obtained from 1 patient.

a S, spinal; IT, infratentorial; ST, supratentorial.

b E, ependymoma; SE, subependymoma; AE, anaplastic ependymoma; MPE, myxopapillary ependymoma; GBM, glioblastoma.

c GTR, gross total resection; STR, subtotal resection.

d DOE, dead of ependymoma; AWD, alive with disease; NED, no evidence of disease; DOD, dead from other disease; DPC, dead of postoperative complications.

e Patient still alive with no evidence of disease progression (August 2013).

M, male; F, female.

**Table II:** Karyotypes and HR-CGH results in 19 ependymoma samples from 18 patients.

Case no.	Karyotype	HR-CGH results
1	68–70,XX,−**X**,−**2**,−**6**,**+7**,**+9**,+10,**+11**,**+14**,**+15**,−**16**[cp4]/46,XX[2]	rev ish enh (5p14p15, **7**, 8q23, **9**, **11**, **14**, **15**) dim (**X**, **2**, **6**, **16**)
2	46,XY	No material available
3	89–94,XXYY,**+2**,−**6**,i**(6)(p10)**[cp11]/46,XY[2]	rev ish enh **(2)**, dim **(6q)**
4	45,XY,−6[6]/44,idem,−Y[16]/46,XY[1]	No material available
5	Culture fail	rev ish dim (17q21, 22q11)
6	64–69,XXY,+Y,−**3**,**+5**,−**6**,**+7**,−**11**,**+13**,−14,+15,−**16**,−**22** [cp6]/46,XY[13]	rev ish enh (Xq21qter, **5**, **7**, 8, **13**) dim (**3**, **6**, **11**, **16p11p13**, **16q12q24**, **22**)
7	69–70,XXY,+Y,**+2**,−**3**,**+5**,**+7**,−**8**,**+12**,−**14**,+15,−**18**,**+20**,−**21**, −**22**[cp7]/46,XY[21]/nonclonal[1]	rev ish enh (Xp11p21, **2**, **5**, **7p13p22**, **7q11qter**, 9p23p24, **12q23**, **12q24**, **20p11pter**, **20q13**) dim (1p36pter, 1q12q21, 1q21, **3**, **8p11p12**, **8p21p23**, **8q21q23**, **8q24**, 9q34, 11q13, **14q32**, 16p13, **18p11**, **18q11q12**, **18q21q22**, 19q13, **21**, **22**)
8	46,XY	No imbalances
9-1	34–36,XY,−**3**,−**6**,−11,−12,−**13**,−**14**,−**15**,−**17**,−**18**,−22[cp6]/46,XY[10]	rev ish enh (2q22q32, 2q34q37, 4q12q21, 4q24q31, 4q32q34, 5p12p14, 5q11q12, 5q21, 5q33q34, 9p23pter, 9q13q21) dim (**3p13p14**, **3p21p25**, **3q12q13**, **3q13q22**, **3q24q27**, **6p12**, **6q22**, **13q12q31**, **14q11q21**, **14q24q32**, **15q15q21, 15q22q24**, **17p11p13, 17q11q21, 17q22q24, 18q12q22**)
9-2	33–36,XY,−3,−6,−10,−11,−12,−13,−14,−18,−22[cp3]	rev ish enh (1p13p34, 1q21qter, 2, 4, 5p, 5q13q31, 5q33q35, 7p13p22, 7q21q22, 7q22q36, 8p21pter, 8q12qter, 9p21pter, 9q22q34, 20p12p13, 20q13, 21q21q22) dim (3p21, 3q27q28, 6p12p23, 6q25, 10p11p14, 10q21, 11p11p15, 11q13, 12p11p12, 12p13, 12q13q14, 12q24, 15q11q24, 17p, 17q11q25, 22q11q13)
10	46,XY [19]	No imbalances
11	46,XY,**der(14)t(2;14)(p23;q22)**[14]/46,XY[1]	rev ish enh (2p23pter) dim **(14q23qter)**
12	44,XY,−**10,del(13)(q22)**,−**22**[12]/44, idem,der(14)t(1;14)(p32;q32)[5]/39–43,idem, −**8**[14],−**11**[9],−**12**[4],−**17**[7],−18[4][cp22]	rev ish enh (7p) dim **(8q11q22, 8q24, 10, 11q13, 12, 13, 17, 22q12qter)**
13	Fail - no metaphases suitable for analysis	No imbalances
14	46,XX [10]	rev ish enh (4, 5, 11, 14, 15, 17, 18, 19q13, 20p11p13, 20q11, 20q13) dim (6p12p22, 6q, 8, 10, 13)
15	81–92,XXX,−X,**del(2)(p13~16p21~23)**,−10,+22[cp12]/46,XX[4]	rev ish dim (1p35p36, **2p16p23**, 16q12, 16q21, 16q24)
16	46,XX[21]	No imbalances
17	45,XY,−**6**[14]/46,XY[4]	rev ish dim **(6)**
18	45,X,−Y[5]/46,XY[19]/nonclonal[4]	No imbalances

Aberrations in bold were detected by both G-banding and HR-CGH.
